# Small-Bowel Capsule Endoscopy in Patients with Suspected Crohn's Disease—Diagnostic Value and Complications

**DOI:** 10.1155/2010/101284

**Published:** 2010-08-05

**Authors:** Pedro Figueiredo, Nuno Almeida, Sandra Lopes, Gabriela Duque, Paulo Freire, Clotilde Lérias, Hermano Gouveia, Carlos Sofia

**Affiliations:** ^1^Department of Gastroenterology, University Hospitals of Coimbra, Avenue Bissaya Barreto, 3000-075 Coimbra, Portugal; ^2^Faculty of Medicine, University of Coimbra, Rua Larga, 3004-504 Coimbra, Portugal

## Abstract

*Background*. The aim of this work was to assess the value of capsule enteroscopy in the diagnosis of patients with suspected Crohn's Disease (CD). *Methods*. This was a retrospective study in a single tertiary care centre involving patients undergoing capsule enteroscopy for suspected CD. Patients taking nonsteroidal anti inflammatory drugs during the thirty preceding days or with a follow-up period of less than six months were excluded. *Results*. Seventy eight patients were included. The endoscopic findings included mucosal breaks in 50%, ulcerated stenosis in 5%, and villous atrophy in 4%. The diagnosis of CD was established in 31 patients. The sensitivity, specificity, positive and negative predictive value of the endoscopic findings were 93%, 80%, 77%, and 94%, respectively. Capsule retention occurred in four patients (5%). The presence of ulcerated stenosis was significantly more frequent in patients with positive inflammatory markers. The diagnostic yield of capsule enteroscopy in patients with negative ileoscopy was 56%, with a diagnostic acuity of 93%. *Conclusions*. Small bowel capsule endoscopy is a safe and valid technique for assessing patients with suspected CD. Capsule retention is more frequent in patients with positive inflammatory markers. Patients with negative ileoscopy and suspected CD should be submitted to capsule enteroscopy.

## 1. Introduction

The current view is that the diagnosis of Crohn's Disease (CD) is established by a combination, not strictly defined, of clinical presentation, endoscopic appearance, radiology, histology, surgical findings, and, more recently, serology [1]. 

The role of small-bowel capsule endoscopy (SBCE) in this context is still debateable [2], namely, because the concept of suspected CD, with implications in the selection of the patients, is itself under discussion. The other reason for debate is the lack of a clear definition of the endoscopic findings that should be considered indicative of CD. Even though lesions such as aphthae, erosions, or ulcers may be considered suggestive of the existence of the disease, the fact is that other aetiologies, namely, the use of nonsteroidal anti-inflammatory drugs (NSAIDs) may also be associated with the presence of these lesions, not forgetting the fact that healthy adults with no history of ingesting pharmaceutical drugs may also present similar lesions [3]. Furthermore, it is not clear whether, in the case of patients with suspected CD, SBCE is superior to other diagnostic methods, namely, ileoscopy [4].

The aim of this study was to assess the value of SBCE in diagnosing CD, as well as the complications associated with the technique.

## 2. Material and Methods

### 2.1. Patients

A retrospective study of patients with suspected CD who had undergone SBCE in a single tertiary care academic centre was carried out. The criteria to perform SBCE in our department include the absence of any clinical or imaging study indicating the existence of stenosis of the small intestine. The following data was collected: age, sex, starting date of symptoms, clinical symptomatology, history of NSAID use, examinations carried out from the onset of complaints to the date of SBCE, endoscopic findings, complications associated with the examination, clinical assessment during the follow-up period, and duration of the same. Patents for whom there were references in the medical files to the use of NSAIDs during the month prior to the examination and patients with a follow-up period of less than six months after the date of the examination were excluded from the study. 

The patients were analysed according to the algorithm proposed by the International Conference on Capsule Endoscopy (ICCE) for suspected CD [5].

Information on patient follow-up was obtained by contacting the referring physician. The diagnosis of CD was established by clinical evaluation during the follow-up period, by a combination of endoscopic, histological, radiological, and/or biochemical investigations [1].

**Figure 1:**
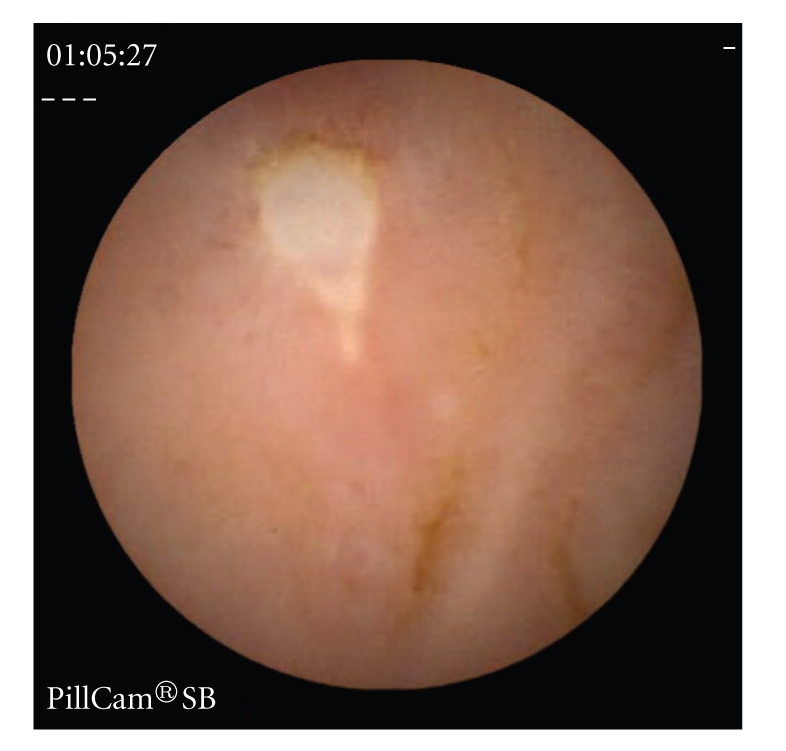
Jejunal ulcer.

**Figure 2:**
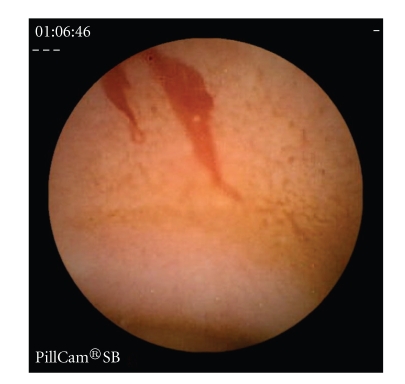
Bleeding jejunal ulcer.

**Figure 3:**
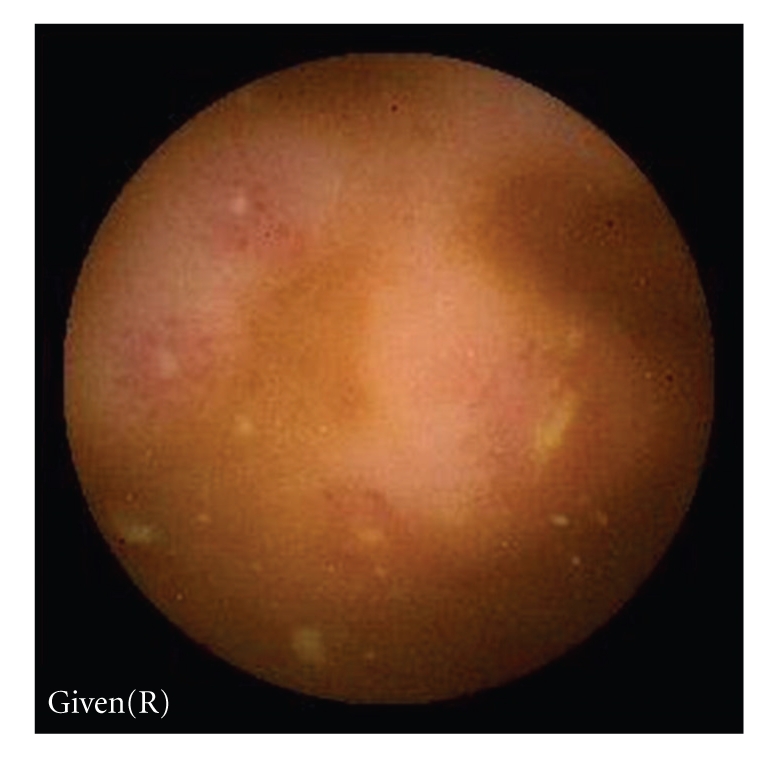
Ileal erosions.

**Figure 4:**
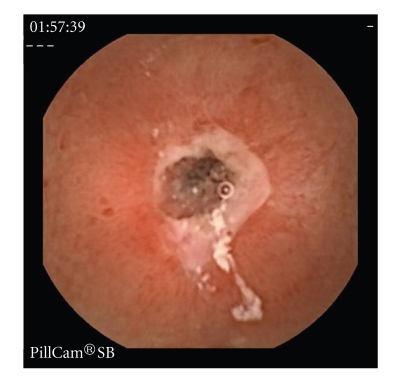
Jejunal ulcerated stenosis.

**Figure 5:**
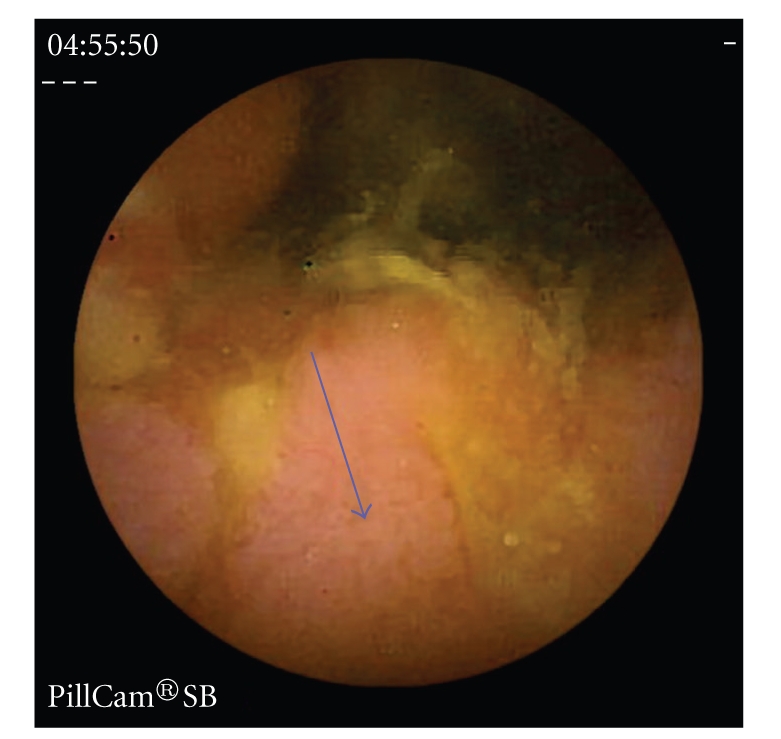
Area of villous atrophy in the ileo (arrow).

Erosions, ulcers, ulcerated stenosis, and villous atrophy were considered suggestive of CD, irrespective of the number of lesions found. Ulcers were defined as white lesions within a crater and with a surrounding erythema (Figures 1 and 2) [6] and erosions as superficial white lesions with surrounding erythema (Figure 3) [6]. The diagnosis of ulcerated stenosis was based on the presence of an ulcer associated with retention of the capsule (Figure 4). The diagnosis of villous atrophy was presumed, but not submitted to histological confirmation, after the endoscopic diagnosis of a circumscribed area of villous denudation (Figure 5).

### 2.2. Capsule Endoscopy Procedure

A PillCam SB (Given Imaging Ltd; Yoqneam, Israel) was used. After an overnight fast of 12 hours, the patients ingested the capsule with a small amount of water with simethicone. No oral purge was administered. All the patients were advised to drink after four hours and, after eight hours, the sensor array and the recording device were removed. The digital video image streams of the examinations were downloaded to the RAPID system. The digital image stream was assessed and interpreted by endoscopy fellows (A.N., L.S., F.P., D.G.) and reviewed by two staff endoscopists (F.P., L.C.). The interobserver agreement was not properly assessed, but all the videos were widely scrutinized and discussed.

### 2.3. Statistical Analysis

The sensitivity, specificity, positive predictive value, negative predictive value, positive and negative likelihood ratio of the diagnostic test, as well as confidence intervals were assessed using the VassarStats Website for Statistical Computation (available at http://faculty.vassar.edu/lowry/VassarStats.html). Statistical comparisons of categorical data were made using the chi-squared test, with the Yates correction when needed, and with the Fisher exact test. A *P* value of less than  .05 was considered significant. The analysis was performed with statistical software (SPSS version 11.5, SPSS Inc, Chicago, Illinois).

## 3. Results

Between January 2001 and December 2007, 95 patients clinically suspected of having CD underwent capsule enteroscopy. Fourteen of the patients were excluded from the study as it was stated that they had taken NSAIDs in the preceding thirty days and a further three were excluded because the follow-up period amounted to less than six months. 

The demographic and clinical characteristics of the remaining 78 patients are showed in Table 1.

With regard to previous endoscopic examinations, all the patients had undergone colonoscopy, but no lesions indicative of CD had been detected in any of the cases. Retrograde ileoscopy had been carried out on 47 patients (60.3%), revealing a slightly congested mucosa in 7 cases. A histological study of the biopsies did not reveal any indications of CD. In 31 patients (39.7%), the intubation of the terminal ileum was not accomplished.

The small-bowel series revealed lesions in 5 (26.3%) of the 19 patients who had undergone enteroclysis and in 5 (10.8%) of the 47 who had undergone small-bowel follow-through (SBFT). A computed tomography (CT) carried out on 37 of the patients revealed lesions in 14 cases (37.8%). 

Seventy eight examinations with capsule were carried out, achieving total enteroscopy in 64 cases (82.1%). Of the 14 cases with incomplete enteroscopy, in 4 (5.1%) this was due to the presence of a stenosis which led to retention of the capsule, whilst in the remaining ten cases it was attributed to slower transit. Six of these patients with incomplete enteroscopy, in which no lesions were detected, were excluded from further analysis, as it was not possible to know if they had any of the findings considered. The remaining four patients, known to present lesions considered suggestive of CD, were included.

No other complications, apart from retention, were recorded. 

Pathological images were detected in 37 patients of the 72 patients considered, giving the technique a diagnostic yield of 51,3%. The main endoscopic findings were mucosal breaks, which were detected in 36 patients (50%). Five of these patients presented ulcers, four of which were in conjunction with erosions, whilst in one case only ulcers were found. The remaining 31 patients presented only erosions. With regard to the number of mucosal breaks, in 3 patients this amounted to 3 or less (4.2%) whereas in 26 cases (36.1%) it totalled 6 or more. After mucosal breaks, the most frequently detected endoscopic findings were ulcerated stenosis, observed in 4 cases (5.5%), followed by areas of villous atrophy, observed in 3 cases (4.2%). In 6 patients, there was more than one type of pathological finding (3 with stenosis and mucosal breaks, and 3 with villous atrophy and mucosal breaks). The remaining 31 patients showed only one type of lesion, namely, an isolated stenosis in one patient and mucosal breaks in the remaining 30 (81% of the patients with lesions). 

We are able to evaluate ICCE criteria in 70 patients (in two there was not enough information available in the patient file) (Table 2). The ICCE criteria were fulfilled in 36 patients (51.4%). Some patients presented, in addition to two gastrointestinal symptoms considered in the algorithm, more than one of the following criteria: extraintestinal manifestations, abnormal imaging studies, or inflammatory markers. 

Among the 36 patients with positive ICCE criteria, 20 (55.6%) presented pathological images on SBCE versus 16 (44.4%) without lesions (*P* = .237). In the subgroup with inflammatory markers, 17 (68%) presented lesions versus 8 (32%), reaching statistical significance (*P* = .022). The presence of ulcerated stenosis was more frequent among patients with ICCE criteria (*P* = .64), the difference being statistically significant only in the subgroup with inflammatory markers (*P* = .014).

During the follow-up period, which lasted on average 28.8 months (sd 13.3 months) (6–65 months), 31 patients (43%) were diagnosed with CD. The demographic and clinical characteristics of these patients are shown in Table 3. In relation to the four patients with ulcerated stenoses which led to retention of the capsule, they presented symptoms for an average of 13 months (4–24 months). Abdominal pain, weight loss, anaemia, and elevated CRP were present in all of them. The prior diagnostic work-up, that included colonoscopy with retrograde ileoscopy in three, SBT in four and CT in one, did not found any lesions. None of the four patients developed symptoms or signs of intestinal occlusion. Two patients underwent surgery involving the resection of a segment of the small intestine, and a histological study of the tissue showed aspects compatible with CD. The other two patients, who were twin brothers, were only given medical treatment and the capsule was expelled voluntarily.

Table 4 shows the sensitivity, specificity, positive predictive value, negative predictive value, positive and negative likelihood ratio of the endoscopic findings, ICCE criteria and ICCE criteria plus endoscopic findings in the diagnosis of CD.

Table 5 shows the capsule findings in patients submitted to retrograde ileoscopy. The negative predictive value for ileoscopy in the diagnosis of CD was 49%. It should be noted that out of the 22 patients subsequently diagnosed with CD in whom ileoscopy had shown no apparent lesions, in 21 cases lesions were revealed during capsule examination. The diagnostic yield for SBCE in the 43 patients who underwent retrograde ileoscopy was 56%, with diagnostic acuity of 93%, 95% sensitivity, 86% specificity, 88% positive predictive value, and 95% negative predictive value.

## 4. Discussion

The sensitivity and, above all, the high negative predictive value and low negative likelihood ratio, suggesting the high probability of absence of the illness in patients who do not show endoscopic lesions, are, in our opinion, the most relevant piece of information to emerge from the study. It is important to emphasize that the methodology used in our study, as with the one reported by Tukey et al. [7], involved a follow-up period which, in our case, lasted more than six months, extending on average to 28.8 months. The CD diagnosis was not, therefore, established immediately on the basis of the capsule enteroscopy findings. We consider this methodology to be more correct, given the recognised difficulties in diagnosing the disease and the absence of a gold standard [1].

The issue of selecting patients for SBCE is of the greatest importance. In fact, the recognition that abdominal pain of an unknown aetiology should not, on its own, constitute an indication for capsule enteroscopy [8, 9], as well as the problem of capsule retention and the high cost of the procedure, must be taken into consideration. Recently, the ICCE issued recommendations about SBCE in cases of suspected CD, formulating an algorithm which proposed that patients who presented suggestive symptoms plus either extraintestinal manifestations, inflammatory markers, or abnormal imaging studies should be selected to undergo capsule enteroscopy [5]. Our results show the high level of success obtained with the technique when this algorithm is used. In this context, it is legitimate to ask whether it would not be preferable, given that the criteria in the aforementioned algorithm can be met, to opt for balloon-assisted enteroscopy, thus preventing any capsule retention and enabling tissue to be collected for biopsy.

A variety of studies have been published seeking to assess the value of SBCE in the diagnostic assessment of patients with suspected CD [6, 7, 10–15]. The inclusion criteria, based on known data relating to the clinical and biological manifestations of CD, are similar, even though the number of patients included in each study varies considerably. It is recognised that, in patients with CD, the endoscopic findings most frequently detected by the capsule are aphthoid ulcers/erosions [16]. However, we are far from reaching a consensus on the number of lesions considered significant. In fact, in the study by Mow et al. [6], the criterion used to presume a diagnosis of CD is the presence of more than three ulcers, whereas in the study by Voderholzer et al. [17] it is the detection of more than ten aphthoid or erosive lesions. The question of NSAID is, naturally, crucial, and it is recognised that NSAID should not be administered to patients involved in these studies for at least thirty days prior to SBCE [18, 19]. Unlike most of the series cited [6, 7, 11, 14, 15], we did not exclude patients from our calculations according to the number of mucosal breaks. In fact, we think that, since there is no consensus on the number of mucosal breaks that should be considered indicative of a diagnosis of CD, the exclusion of patients on the basis of a numerical criterion appears arbitrary. It should be emphasised that in our study only three patients presented three or fewer mucosal breaks. This data is relevant, given that in the work of Goldstein et al. [3] no healthy individual without a history of ulcerogenic medications presented more than three mucosal breaks. 

This study highlights the good diagnostic yield of the technique in patients with suspected CD. In fact, diagnostic yield achieved 51.3% in our series, which places it within the range spanning the 37.5% cited in the study by Mow et al. [6] and the 70.6% of Fireman et al. [10]. These variations may be related to the different admission criteria associated with NSAID use, together with the different characteristics of the patients included in the various studies.

The rate of incomplete enteroscopies, which in our series was 17.9%, is similar to the rates reported in the literature considering all the indications [20, 21], consistent with the hypothesis that CD may not increase the risk of an incomplete observation [21]. The capsule retention rate in our series is higher than the one reported in studies which include all indications [20, 22]. When weighing against series which include patients with suspected CD, the rate is also higher, namely, when compared with the rate reported by Cheifetz et al. [23] and by Li et al. [22], 1.6% and 0%, respectively. It should be noted that for none of our patients did the clinical assessment and/or the prior imaging studies suggest the presence of stenosis, and this conforms to reports in other studies which demonstrate the low reliability of clinical and imaging studies in predicting the existence of stenoses [22, 24]. In this context, the recommendation to carry out imaging studies before capsule enteroscopy aiming the exclusion of a stenosis [5, 25] is hard to understand. In fact, we found that ulcerated stenosis leading to capsule retention were significantly more frequent in the subgroup of patients with positive inflammatory markers. It should be emphasised that none of our four patients with retained capsules developed any clinical manifestations indicative of intestinal occlusion and only two underwent surgery. 

The retrospective nature of our study, along with the fact that many patients included are referred from other hospitals, may explain the high rate of unaccomplished intubations of the terminal ileum. Nevertheless, the question of ileoscopy in the diagnostic investigation of these patients also merits discussion. The aim of this study was not to compare ileoscopy with SBCE, given that patients who presented lesions indicative of CD in the terminal ileum did not undergo SBCE. In this context, it was only possible to calculate the negative predictive value of the ileoscopy which, as it amounted to 49%, attests to the fact that when the ileoscopy is negative in these patients, the probability of existence of the disease is high. Furthermore, the diagnosis of CD was later established in 22 (51.2%) of the 43 patients who had a negative ileoscopy, and that in 21 of these SBCE revealed lesions. The SBCE diagnostic yield for patients with a negative ileoscopy was 56%, with a high diagnostic acuity, sensitivity, and negative predictive value for the diagnosis of CD. The meta-analysis published by Triester et al. [4] showed that SBCE was not significantly better than ileoscopy for patients with suspected CD. However, a study of double-balloon enteroscopy demonstrated that, in a high percentage of patients, ileal involvement in CD may be outside the range of the ileoscopy [26]. In fact, the most recent published meta-analysis by Dionisio et al. shows that SBCE is superior to colonoscopy with ileoscopy [27]. Our results corroborate this finding, signifying that, faced with clinically suspected CD and a negative ileoscopy, the use of enteroscopy, namely, with capsule, is advisable.

In conclusion, SBCE is a valid diagnostic tool in patients with suspected CD, namely, when inflammatory markers are present. It is particularly informative when lesions are not detected, a case in which the diagnosis of CD is very unlikely. The use of SBCE in this indication may lead to retention of the capsule, more frequently in the subgroup of patients with positive inflammatory markers, but this event is not accompanied by symptoms of intestinal occlusion and can be remedied without the need for surgery. Finally, the diagnosis of CD is not infrequent among patients with negative ileoscopy, suggesting that capsule enteroscopy is advisable in these cases.

## Figures and Tables

**Table 1:** Demographic and clinical characteristics of the patients.

Number of patients	78
Age (years) (mean ± SD)	37.2 ±16.4
Gender (female) *n* (%)	53 (67.9%)
Abdominal pain *n* (%)	62 (79.5)
Diarrhoea *n* (%)	47 (60.3)
Weight loss *n* (%)	27 (34.6)
Arthralgias *n* (%)	27 (34.6)
Fever *n* (%)	11 (14.1)
Duration of symptoms (months) (mean ± SD)	22.3 ± 26.2
Anaemia *n* (%)	42 (53.8)
Elevated CRP *n* (%)	28 (35.9)

SD: standard deviation; CRP: C-reactive protein.

**Table 2:** Distribution of patients according to ICCE criteria.

	*N* (%)
ICCE criteria present	36 (51.4)
Symptoms plus extraintestinal symptoms and signs	19 (27.1)
Symptoms plus abnormal imaging*	10 (16.9)
Symptoms plus inflammatory markers	25 (35.7)
ICCE criteria absent	34 (48.6)

Total	70

*This group includes only the 59 patients submitted to CT or small bowel series.

**Table 3:** Demographic and clinical characteristics of the 31 patients with confirmed CD during the follow-up.

Age (years) (mean ± SD)	35.8 ± 16.2
Gender (female) *n* (%)	20 (64.5%)
Abdominal pain *n* (%)	23 (74.2)
Diarrhoea *n* (%)	47 (60.3)
Weight loss *n* (%)	17 (54.8)
Arthralgias *n* (%)	7 (22.6)
Fever *n* (%)	7 (22.6)
Duration of symptoms (months) (mean ± SD)	18.5 ± 17.2
Anaemia *n* (%)	20 (64.5)
Elevated CRP *n* (%)	16 (51.6)
ICCE criteria present *n* (%)	20 (68.9)
Symptoms plus extraintestinal symptoms and signs *n* (%)	6 (19.4)
Symptoms plus abnormal imaging *n* (%)	9 (37)
Symptoms plus inflammatory markers *n* (%)	17 (58.6)
Suggestive endoscopic findings present *n* (%)	29 (93.5%)
Erosions/ulcers *n* (%)	29 (100)
Ulcerated stenosis *n* (%)	4 (13.7)
Villous atrophy *n* (%)	3 (10.3)
Duration of follow-up (months) (mean ± SD)	30.7 (13.2)

SD: standard deviation; CRP: C-reactive protein.

**Table 4:** Value of different criteria in the diagnosis of CD.

	Sens. (95%CI)	Spec. (95%CI)	PPV (95%CI)	NPV (95%CI)	LLR+ (95%CI)	LLR− (95%CI)
Lesions on SBCE	93 (75–98)	80 (64–90)	77 (59–88)	94 (79–99)	4.7 (2.5–8.9)	0.08 (0.02–0.3)
ICCE criteria	65 (45–81)	58 (42–73)	52 (35–69)	70 (52–84)	1.5 (1–2.4)	0.58 (0.34–1)
plus lesions on SBCE	100 (70–100)	86 (64–96)	85 (61–86)	100 (79–100)	7.3 (2.5–20)	0
Symptoms plus extraint.symptoms/signs	24 (11–43)	70 (54–83)	36 (17–61)	56 (42–70)	0.8 (0.3–1.8)	1.0 (0.8–1.34)
plus lesions on SBCE	77 (40–96)	100 (80–100)	100 (50–100)	91 (70–98)	(a)	0.22 (0.06–0.7)
Symptoms plus abnormal imaging	29 (13–51)	91 (75–97)	70 (35–91)	65 (50–77)	3.4 (0.9–11)	0.77 (0.59–1)
plus lesions on SBCE	100 (46–100)	96 (80–99)	83 (36–99)	100 (84–100)	29 (4,2–198)	0
Symptoms plus Inflammatory markers	55 (35–73)	78 (61–88)	64 (42–81)	71 (55–83)	2.5 (1.2–4.8)	0.57 (0.37–0.8)
plus lesions on SBCE	88 (863–98)	96 (78–99)	94 (69–99)	92 (74–98)	23.1 (3.3–159)	0.1 (0.03–0.4)

Sens.: sensitivity; Spec.: specificity; PPV: positive predictive value; NPV: negative predictive value; LLR+: likelihood ratio positive; LLR−: likelihood ratio negative; CI: Confidence Interval; extraint.: extraintestinal; ^(a)^infinity.

**Table 5:** Endoscopic lesions detected by SBCE in patients with negative retrograde ileoscopy and subsequent diagnosis of CD.

Negative ileoscopy (*N* = 43)			
	CD confirmed	CD not confirmed	Total
Endoscopic lesions present, *n* (%)	21 (87.5)	3 (12.5)	24 (100)
Endoscopic lesions not present, *n* (%)	1 (5.3)	18 (94.7)	19 (100)

Total *n* (%)	22 (51.2)	21 (48.8)	43 (100)
